# First Aid Willingness Questionnaire for Schoolchildren: An Exploratory Factor Analysis and Correlation Study

**DOI:** 10.3390/children9070955

**Published:** 2022-06-25

**Authors:** Zsolt Katona, Klára Tarkó, Tamás Berki

**Affiliations:** 1 Institute of Physical Education and Sport Sciences, Juhász Gyula Faculty of Education, University of Szeged, 6725 Szeged, Hungary; katona.zsolt@szte.hu; 2 Institute of Applied Health Sciences and Environmental Education, Juhász Gyula Faculty of Education, University of Szeged, 6725 Szeged, Hungary; tarko.klara@szte.hu; 3 MTA-SZTE Health Promotion Research Group, 6725 Szeged, Hungary

**Keywords:** first aid, adolescent, sport, injury, attitude, first aid willingness

## Abstract

The goal of this study was to explore the factor structure of the First Aid Willingness Questionnaire and determine its correlations and associations between sociodemographic and sport-related variables. A total of 413 adolescents participated in this study (mean age = 14.2 years). They consisted of 221 boys and 193 girls. Besides sociodemographic and sport-related questions, the First Aid Willingness Questionnaire was used to understand the student’s first aid attitudes. The exploratory factor analysis revealed a four-factor model. The first factor was named first aid willingness for peers, which includes willingness to help friends and family members. The second factor contained factors to help strangers; thus, it was named first aid willingness for strangers. The analysis revealed a third factor that assessed the students’ knowledge of first aid. The last factor contained the students’ negative emotions. The correlation between the factors showed that knowledge had a positive association with all the other factors. Adolescents’ willingness to help their peers was highly associated with helping strangers, but negative emotions had a negative correlation with helping unknown people. Sport-related variables were investigated to determine the effects on first aid attitudes. Even though sport seemed to increase first aid willingness, future studies need to explore its associations. We believe that a deeper understanding of this topic could help prevent serious injuries or death in emergencies.

## 1. Introduction

As formulated by Cuttle, Pearn, McMillan, and Kimble [1] “First aid is emergency care or treatment given before regular medical aid can be obtained” (p. 768). Anyone can apply it to others or even to themselves, with the main goal of saving a life, preventing further injuries or illnesses, and aiding recovery [2]. The provision of first aid requires recognition, assessment, and prioritization, being aware of one’s competencies and limitations, and asking for extra help if it is needed [2]. Even though the importance of first aid is widely known, the rate of quality first aid offered by laypeople is generally low worldwide [3]. However, training in first aid could increase the willingness for initial care. A broad range of studies agree that teaching first aid and cardiopulmonary resuscitation (CPR) to children and adolescents could increase the willingness and quality of help for individuals in emergency situations [4,5]. Previous studies have also indicated that knowledge and attitudes are important elements of first aid willingness [6,7], which could be increased by conscious skill development and proper first aid training.

Several studies have investigated attitudes and knowledge in first aid [8,9,10,11]. These studies generally concluded that knowledge is an essential factor in providing first aid, which could be introduced to children at an early age. Studies have shown that 5–18-year-old children and adolescents who learn first aid are also willing to provide help if necessary [11,12,13]. Furthermore, previous studies have found that school-age children are more likely to engage in CPR or first aid training than older individuals [14]. A recent study showed that teaching schoolchildren first aid could result in a more positive attitude toward bystander CPR [15]. There are also other additional benefits of first aid training. It was found to be useful for increasing children’s and adolescents’ confidence and self-efficacy [13]. Due to the positive effects of first aid, medical professionals worldwide suggest increasing the number of school programs or enhancing their integration into school curricula [6,16].

First aid knowledge also affects first aid attitudes. For example, previous studies found that pupils with lower knowledge were less likely to attempt to resuscitate a stranger and were less motivated to learn more about CPR than those with positive attitudes [14]. Furthermore, Lee [17] revealed that Korean teachers refused to have pupils with epilepsy in their classes because they were afraid of the situation of acting on a seizure crisis. Individuals are affected by positive and negative emotional responses during an accident. That includes, for example, feelings of sadness, fright, concern, relief, hope, and pride [18]. Researchers also identified the factors behind these feelings, such as serious injuries [19], the victim being a stranger [13], dirty casualty [20], and the fear of hurting the victim [13,20] could cause negative feelings, while other factors, such as the victim being a relative, a friend, or a child, could positively influence the attitude and behavior toward first aid [13,14,20]. Overall, it seems that the willingness to provide first aid is dependent on the individual’s knowledge and positive and negative attitudes, which are mainly influenced by the appearance of the victim.

The determinants that influence first aid behavior are clear, but there are some validated questionnaires in the international literature to assess this issue. Pei-Chuan [21] investigated attitudes and willingness toward bystander CPR with a 14-item questionnaire. De Smedt [22] investigated knowledge, attitude, and experience toward CPR and AED (Automated External Defibrillator) among Flemish schoolchildren. Similar factors were used in the Pei [23] study in the contest of bystander traffic accidents. Outside of these instruments, there are limited existing surveys and currently no validated Hungarian questionnaires to investigate first aid attitudes and knowledge.

Previous studies have shown that first aid attitudes and knowledge can be differentiated by age, gender, education, and family background [24,25,26]. There are also extracurricular activities that could promote first aid behavior. Physical activity and sports have a wide range of health and mental benefits, such as the prevention of cardiovascular disease, weight control, and even an increase in school motivation and subjective well-being [26,27,28,29,30]. Furthermore, sport has been identified as an activity that could increase knowledge and attitudes in first aid [31]. Gabbe [32] found that injury or accident prevention is higher among athletes than among people engaged in any other activity. Celse et al. [33] found that athletes exhibited more cooperative behavior than non-athletes. Furthermore, we should acknowledge that many athletes in their sports learn to provide assistance to their peers in a new element or technique [34]. We believe it might positively affect their first aid attitude, but this relationship has not yet been investigated. Since there are only a few studies about the role of sports participation in first aid behavior, it should be further investigated.

Due to the importance of first aid, the main goal of this study was to introduce a questionnaire that could identify individuals’ first aid knowledge and attitudes. In addition, our goal is to determine the age- and gender-related differences and the effect of sports participation on first aid knowledge and attitudes. We hypothesized the following: (1) Our First Aid Willingness Scale has four factors; (2) First aid knowledge and attitudes are different by gender and age; (3) Previous first aid experience increases first aid knowledge and attitudes; and (4) Sports participation could increase the level of first aid knowledge and attitudes.

## 2. Materials and Methods

### 2.1. Participants and Procedure

Data collection was conducted in the 2018/19 school year in Szeged, Hungary. Six different schools were involved in this study. The research was authorized by the school principals. Parents and pupils were informed by schoolteachers in email about the goals of the research, and written informed parental consent was obtained. The Hungarian questionnaire was self-administered, anonymous, and voluntarily. Using a paper-and-pencil method, the participants filled out the questionnaires in physical education classes supervised by PE teachers. Completion took approximately 10–15 min. Participants were assured that there were no right or wrong answers. Ethical approval was obtained from the university’s Institutional Review Board (Ethical Approval Number: 2/2018-SZTE).

A total of 414 adolescents participated in this study, with an age range of 13 to 16 years (mean_age_ = 14.2 years, SD_age_ = 1.08 years). The sample consisted of 221 boys (52.9%) and 193 girls (47.1%). The participants reported their previous experience with first aid (e.g., in school classes or sport activities). Of the sample, 61.4% (n = 255) had previous experiences with first aid, and 38.4% (n = 159) did not have any experience with first aid. The participants were asked to describe their extracurricular sports activities. However, the adolescents named 20 different sports; 40.2% (n = 166) of them did not participate in any sports activities or only for one day. Of the sample, 35% (n = 145) reported that they exercise 2–3 times a week, and 24% (n = 103) of the adolescents had physical training sessions 4–5 times a week. The preferred types of sports could be grouped as team sports (31.7%, n = 131) and individual sports (28.0%, n = 117). We asked the students to rate the physical activity levels of their families. Of the sample, 41.6% (n = 170) reported a high level of physical activity, 26.2% (n = 107) reported a low level of physical activity, and 32.3% (n = 137) of the participants reported that their families did not do any physical activity at all.

### 2.2. Measures

#### 2.2.1. Sociodemographic and Sport-Related Questions

Sociodemographic data included the students’ age, gender, family background, and the characteristics of their extracurricular sport activities (e.g., How many times a week do you exercise? What sport do you practice?), and their previous experiences with first aid (e.g., “Have you learned any first aid skills?”). The level of the family’s physical activity was measured with a single question (“How physically active is your family?”). The answered categories were as follows: none (not participating at all in any sport activities), low (sometimes participating in sport activities such as hiking, biking, etc.), and high (participating in regular sport activities such as hiking, biking, etc.).

#### 2.2.2. Development of the First Aid Willingness Questionnaire

The First Aid Willingness Questionnaire was based on the literature findings. As was seen earlier, knowledge and attitudes are the main contributors to first aid willingness [13,14,17,18,19,24]. Furthermore, the willingness to receive first aid could depend on the appearance of the victim [13,20]. Eighteen scenarios were created, and they were grouped into four topics based on previous findings: first aid willingness when family and friends are involved (e.g., My brother falls while skating. I help him immediately.); first aid willingness when strangers are concerned (e.g., An old man faints in the street. I notice him and call for help.); knowledge (e.g., An adult woman lies unconscious in the corner. I know how to perform CPR.); negative emotions (e.g., I would be afraid to help someone lying unconscious). The panel of experts, including the schoolteacher, was asked to read and rate the questions. Then, a group of 10 students responded to the survey individually. They were asked if the items were clear and understandable. Based on their recommendations, some questions were rephrased to make them clearer for the adolescent age group. The finalized questionnaire was then administered to the participants, who were asked to express their attitudes about the listed scenarios with the help of a five-point Likert-type scale (1 = Not at all typical of me, 5 = Completely typical of me).

### 2.3. Statistical Analysis

SPSS for Windows 23.0 statistical software was used to analyze the data. Besides descriptive statistics (e.g., mean, standard deviations), exploratory factor analysis (EFA) with the maximum likelihood method was used to identify the possible factors of the First Aid Willingness Questionnaire. According to the Kaiser criterion, factors with an eigenvalue of more than 1 were included in this study. The internal consistency of the factors was assessed using Cronbach’s alpha. Furthermore, correlation analyses (Pearson’s coefficients) were used to assess the bidirectional relationships among the EFA factors. Finally, significant differences were explored according to these factors. Hence, an independent sample *t*-test was used on gender, age, first aid experience, and sport type. One-way ANOVA was used for the family sport and weekly sport participation variables.

## 3. Results

### 3.1. Characteristic of the Questionnaire

Table 1 displays the mean, standard deviation, skewness, kurtosis, and range for the items of the First Aid Willingness Questionnaire. The mean of the scale was 3.66 (SD = 0.64). The highest score on the questionnaire was obtained for item twelve (M = 4.41; My brother falls while skating. I help him immediately). The lowest score was obtained for item three (M = 2.44; A girl in art class cuts her finger, which is bleeding. I’m very frightened.) The skewness and kurtosis values were acceptable (−1.5, 1.5) except for items six and twelve [35].

### 3.2. Exploratory Factor Analysis of the Questionnaire

Exploratory factor analysis (EFA) was used to identify the factor structure of the First Aid Willingness Questionnaire (Table 2). The final factor structure revealed four factors and included 16 items, with a minimal loading of 0.40. The factors accounted for 56.26% of the variance, and the Kaiser–Mayer–Olkin index was 0.87. The first factor included five items about willingness to help friends and family members. Therefore, it was named ‘first aid willingness for peers. The factor had an eigenvalue of 4.75 and explained 29.73% of the variance. The second factor contained four items about helping strangers in an emergency. The eigenvalue was 2.07, with a 12.99% variance, and the factor was named ‘First aid willingness for strangers. The third factor accounted for 7.19% of the variance and had an eigenvalue of 1.15. It included four items and was named ‘negative emotions’ since it contained feelings such as fear, panic, and fright. The last factor contained items about CPR, bandage, and recovery position; therefore, it was named ‘knowledge’. The factor had an eigenvalue of 1.01 and explained 6.34% of the variance. The internal consistency of the scales showed acceptable reliability since it varied between 0.70 and 0.77. Two items were deleted for statistical reasons (item 6, item 12).

### 3.3. Correlations between the Questionnaire’s Factors

Table 3 shows the factor correlation with the mean and standard deviation. Positive associations were found between ‘first aid willingness for peers’ and ‘first aid willingness for strangers’ and ‘knowledge’. ‘First aid willingness for strangers’ was negatively associated with ‘negative emotions’ and positively associated with ‘knowledge’. ‘Negative emotions’ had a negative relationship with ‘knowledge’. The highest mean score of the subscale was for the ‘first aid willingness for peers’ factor, and the lowest score was for the ‘negative emotions’ factor.

### 3.4. Factor Differences between the Subscales of the First Aid Willingness Questionnaire

In the final part of our analysis, we investigated the relationship between the identified factors, the sociodemographic (gender, age) variables, the first aid background of participants, and the sport-related variables (sport type, family sport, weekly sport). Girls had a significantly higher willingness to provide first aid for their peers than boys (Table 4). Significant differences were found in knowledge among the age groups. Older students (age = 13–14) had more knowledge than younger students (age = 15–16). Those participants who had previous first aid experience reported a significantly higher level of first aid willingness for peers, first aid willingness for strangers, and knowledge. Sport also seems to affect first aid attitudes. The students participating in any sports with a weekly frequency of 4–5 times reported a significantly higher first aid willingness for peers, first aid willingness for strangers, and knowledge. A higher level of family sport showed a significant difference in first aid willingness for peers and knowledge. The type of sport did not affect first aid attitudes in our sample.

## 4. Discussion

The goal of this study was to explore the factor structure of the newly created First Aid Willingness Questionnaire. Furthermore, we investigated the factor relationships between sociodemographic variables and sports participation. Our results showed four independent factors that assess willingness to first aid for friends and strangers, negative emotions, and knowledge. After correlation analysis, it was found that knowledge is an important factor for first aid willingness since it was associated with help for peers and strangers. However, a lower level of knowledge is associated with negative emotions. Gender, age, and sports were found to be mediator variables on the factors of the First Aid Willingness Questionnaire.

The exploratory factor analysis showed a four-factor model that helps understand first aid behavior among adolescent children. Sixteen items were included in the final structure. The factors were named as ‘first aid willingness for peers’, ‘first aid willingness for strangers’, ‘negative emotions’, and ‘knowledge’. Previous studies showed differences in the individual’s behavior when they provided help to a friend or a stranger [14,36]. Thus, our result was expected, and it is consistent with our hypothesis. ‘Negative emotions’ is an important factor in considering first aid attitude since children often get frightened in an emergency. Previous studies have shown that first aid provision is stressful and involves a negative psycho-emotional transition [3,37]. It is important to keep negative feelings as low as possible to increase the first aid attitude. One way to prevent negative feelings is to improve education on first aid. Our last factor was measuring the individual’s knowledge of performing first aid. Other research has also shown the importance of first aid knowledge [14,16,17]. It should be noted that professionals and researchers all over the world highlighted that first aid knowledge should be included in school curricula since it is associated with first aid attitude [15].

The correlation analysis was consistent with previous findings. All the studies highlighted the importance of first aid knowledge [26,38,39]. In our study, we found that children with a higher level of first aid knowledge are more willing to help others (peers and strangers as well) and perceive fewer negative feelings in an emergency. This is consistent with other studies investigating this issue [7,14,40]. Our results also showed that children with higher negative emotions have a negative attitude about giving first aid to unknown people. We believe that the attitude behind this result is twofold. First, the children had less knowledge of first aid. Second, the pupils might feel uncomfortable during a first aid situation. De Buck and his colleagues [16] collected factors that negatively influenced the help provided by children. Factors like failing as a rescuer, fear of hurting the victim, fear of disease transmission, a dirty casualty (vomit, an unpleasant smell), bleeding, serious injuries, dangers to the rescuer, the victim being a stranger, or drug user, being in a public place, and fear of being sued were identified ([16]; p. 10). There is another association that should be highlighted. First aid willingness for peers and strangers is highly correlated. This means that children who are willing to help their friends and family are more likely to help unknown people.

In the final part of this study, we tested our four-factor questionnaire using sociodemographic and sports variables. We found that girls were more willing to help their peers than boys. The reason behind this result might be that girls in this age group are more caring and have more empathy than boys [41]. The older age group (age = 15–16) had a higher level of knowledge than the younger children. It was interesting to see the importance of the previous first aid training since it significantly affected our factors (except negative emotions). The association between knowledge and age was expected and was consistent with previous findings [42,43]. As hypothesized, previous first aid experience increased knowledge and attitude. These results are in line with those studies that suggest that first aid training needs to be integrated into school curricula [16].

Only a few studies have investigated the effects of sports participation on first aid [32,39]. However, we believe the nature of sports could help to increase first aid attitudes, since athletes must learn to prevent sports injuries and help others if these occur. Moreover, athletes should assist each other in performing the different elements and techniques [34], which might give them a different perspective on helping. We believe that our findings on sport participation frequency strengthen this assumption. Our results indicate that athletes with higher involvement in their sports have more knowledge and are more willing to help their friends and strangers. Our results showed that not only individual sports but also families with more sport involvement might experience more situations (e.g., injuries) where first aid is needed; therefore, they are more prepared for emergencies. Nevertheless, future studies should investigate its associations more deeply using new variables and other perspectives. For example, sports can increase confidence and self-efficacy, which is correlated with first aid attitude; thus, sport might have a bigger role in first aid attitude [13,44].

The study has some limitations that need to be emphasized. First, the First Aid Willingness Questionnaire is not a validated scale. Even though the Cronbach’s alpha values were good, the reliability of this scale needs to be increased. The second issue is that generalizability is limited due to convenience sampling. We must acknowledge that the self-administered questionnaire is a limitation too since we cannot get a realistic picture of the behavior of the children. Our future goal is to remove these limitations, as we are planning to modify the scale to increase its reliability. In future studies, we would like to validate the questionnaire. Our goal is to understand more deeply the effects of first aid training among children, athletes, and young adults; this new variable will be used in future studies. Hopefully, we can get a deeper understanding of the associations between first aid attitude and knowledge.

## 5. Conclusions

In summary, we found a four-factor structure in our First Aid Willingness Questionnaire, as hypothesized. The associations between the factors showed that knowledge plays a role in all the other factors. Children’s willingness to help their peers is highly associated with helping strangers, but negative emotions have a negative correlation with helping unknown people. Sociodemographic and sports participation variables seem to be associated with first aid attitudes and behaviors, but future studies need to investigate these connections.

We believe our study could be useful for professionals, teachers, and researchers who strive to understand the factors behind first aid attitudes. We think a deeper understanding of this topic could help prevent serious injuries or death in emergencies.

## Figures and Tables

**Table 1:** Mean, SD, skewness, and kurtosis of the First Aid Willingness Questionnaire.

Item	M	SD	Skewness	Kurtosis	Range
1. My friend is injured on the playground. I help him immediately.	3.82	0.94	−0.63	0.06	1–5
2. I see a bleeding head. I know how to apply a pressure bandage.	3.83	0.93	−0.68	0.1	1–5
3. My classmate cut his finger, and it bled. I became anxious.	2.44	1.21	0.58	−0.60	1–5
4. An old man faints in the street. I notice him, and I call for help.	3.97	0.86	−0.61	0.08	1–5
5. My cousin seems to break his arm. I help him immediately.	3.91	0.88	−0.60	0.08	1–5
6. My brother has an accident with a bike. I help him immediately.	4.34	0.93	−1.60	2.23	1–5
7. The old lady next door is lying unconscious on the ground. I do not know what to do.	2.7	1.13	0.32	−0.68	1–5
8. A homeless person is lying unconscious on the street. I help him immediately.	3.45	1.05	−0.49	−0.24	1–5
9. Someone at the school is lying on the floor. I walk there, and ask how he is.	3.91	0.89	−0.79	0.43	1–5
10. A woman lies unconscious in the corner. I know how to perform CPR.	3.54	1.01	−0.39	−0.30	1–5
11. Two of my classmates were injured before class. I ask for help immediately.	4.15	0.94	−1.31	1.45	1–5
12. My brother falls while skating. I help him immediately.	4.41	0.94	−1.75	2.76	1–5
13. Two unknown cyclists collide. I help them and ask for help if necessary.	3.92	0.9	−0.73	0.47	1–5
14. A boy fell out of the swing in the playground. I help him immediately.	4.03	0.92	−0.87	0.51	1–5
15. In the case of a street accident, I know how to ask for medical help.	3.44	0.96	−0.26	−0.20	1–5
16. If I see someone injured, I help him immediately.	3.83	0.97	−0.60	0.05	1–5
17. I would be afraid to help someone unconscious.	2.95	1.15	0.02	−0.88	1–5
18. In the case of a serious accident in my neighborhood. I would be panicking.	3.16	1.2	−0.10	−0.91	1–5

**Table 2:** Final factor structure of the First Aid Willingness Questionnaire.

	Factors			
	1. (First Aid Willingness for Peers)	2. (First Aid Willingness for Strangers)	3. (Negative Emotions)	4. (Knowledge)
Item 11	0.75			
Item 9	0.72			
Item 5	0.65			
Item 14	0.6			
Item 1	0.56			
Item 4		0.83		
Item 13		0.63		
Item 16		0.57		
Item 8		0.55		
Item 18			0.75	
Item 7			0.73	
Item 3			0.69	
Item 17			0.68	
Item 15				0.76
Item 10				0.75
Item 2				0.7
Eigenvalue	4.75	2.07	1.15	1.01
% of variance	29.73	12.99	7.19	6.34
Cronbach alpha	0.77	0.72	0.7	0.7

**Table 3:** Bivariate correlation among the factors with factor mean and standard deviation.

Factors	1	2	3	4
1. First aid willingness for peers	1			
2. First aid willingness for strangers	0.60 ***	1		
3. Negative emotions	−08	−0.11 *	1	
4. Knowledge	0.37 ***	0.48 **	−0.13 **	1
Mean	3.96	3.79	2.88	3.6
SD	0.66	0.7	0.84	0.76

Note. * *p* < 0.05; ** *p* < 0.01; *** *p* < 0.001.

**Table 4:** Differences between the factors of the First Aid Willingness Questionnaire by sociodemographic and sport variables.

	First Aid Willingness for Peers	First Aid Willingness for Strangers	Negative Emotions	Knowledge
Gender				
Boy	3.87 (0.63)	3.77 (0.67)	2.77 (0.84)	3.60 (0.80)
Girl	4.07 (0.67)	3.81 (0.73)	2.85 (0.85)	3.59 (0.71)
*t-value*	−3.01 ***	−0.65	−0.95	0.21
Age				
13–14	3.95 (0.66)	3.75 (0.70)	2.80 (0.87)	3.51 (0.79)
15–16	3.98 (0.67)	3.85 (0.69)	2.81 (0.80)	3.75 (0.69)
*t-value*	−0.49	−1.43	−0.12	−3.15 ***
First aid experience				
Yes	4.08 (0.60)	3.87 (0.67)	2.77 (0.88)	3.71 (0.75)
No	3.73 (0.72)	3.62 (0.73)	2.89 (0.76)	3.38 (0.75)
*t-value*	5.17 ***	3.53 ***	−1.29	4.10 ***
Sport type				
Team	4.06 (0.59)	3.82 (0.71)	2.82 (0.87)	3.64 (0.89)
Individual	3.96 (0.73)	3.85 (0.78)	2.75 (0.89)	3.64 (0.70)
*t-value*	1.1	−0.26	0.6	0.92
Family sport				
None	3.91 (0.66)	3.72 (0.68)	2.89 (0.83)	3.48 (0.74)
Low	3.87 (0.71)	3.73 (0.71)	2.72 (0.89)	3.61 (0.69)
High	4.06 (0.61)	3.88 (0.70)	2.80 (0.85)	3.70 (0.80)
*F-value*	3.26 *	2.54	1.35	3.16 *
Sport frequency				
0–1 day	3.82 (0.70)	3.67 (0.69)	2.81 (0.85)	3.52 (0.76)
2–3 day	4.00 (0.60)	3.81 (0.63)	2.71 (0.92)	3.59 (0.74)
4–5 day	4.15 (0.62)	3.96 (0.76)	2.80 (0.84)	3.74 (0.78)
*F-value*	8.50 ***	5.54 **	0.83	3.71 *

Note. * *p* < 0.05; ** *p* < 0.01; *** *p* < 0.001.

## Data Availability

The data presented in this study are available on request from the corresponding author.

## References

[1] Cuttle L., Pearn J., McMillan J.R., Kimble R.M. (2009). A Review of First Aid Treatments for Burn Injuries. Burns.

[2] Singletary E.M., Zideman D.A., Bendall J.C., Berry D.C., Borra V., Carlson J.N., Cassan P., Chang W.-T., Charlton N.P., Djärv T. (2020). 2020 International Consensus on First Aid Science With Treatment Recommendations. Circulation.

[3] Tannvik T.D., Bakke H.K., Wisborg T. (2012). A Systematic Literature Review on First Aid Provided by Laypeople to Trauma Victims. Acta Anaesthesiol. Scand..

[4] Connolly M., Toner P., Connolly D., McCluskey D.R. (2007). The ‘ABC for Life’ Programme—Teaching Basic Life Support in Schools. Resuscitation.

[5] Plant N., Taylor K. (2013). How Best to Teach CPR to Schoolchildren: A Systematic Review. Resuscitation.

[6] Katona Z. (2018). Attitudes of university students towards First Aid and CPR. Pilot study with online questionnaires measuring training and performing First Aid and CPR. Arena-J. Phys. Act..

[7] Wilks J., Kanasa H., Pendergast D., Clark K. (2016). Emergency Response Readiness for Primary School Children. Aust. Health Rev..

[8] Joseph N., Narayanan T., bin Zakaria S., Venugopal Nair A., Belayutham L., Mihiraa Subramanian A., Gopakumar K.G. (2015). Awareness, Attitudes and Practices of First Aid among School Teachers in Mangalore, South India. J. Prim. Health Care.

[9] Petrić J., Malički M., Marković D., Meštrović J. (2013). Students’ and Parents’ Attitudes toward Basic Life Support Training in Primary Schools. Croat. Med. J..

[10] Thornberg R. (2007). A Classmate in Distress: Schoolchildren as Bystanders and Their Reasons for How They Act. Soc. Psychol. Educ..

[11] Bollig G., Wahl H.A., Svendsen M.V. (2009). Primary School Children Are Able to Perform Basic Life-Saving First Aid Measures. Resuscitation.

[12] Jimenez-Fabrega X., Escalada-Roig X., Miro O., Sanclemente G., Diaz N., Gomez X., Villena O., Rodriguez E., Gaspar A., Molina J.E. (2009). Comparison between Exclusively School Teacher-Based and Mixed School Teacher and Healthcare Provider-Based Programme on Basic Cardiopulmonary Resuscitation for Secondary Schools. Emerg. Med. J..

[13] Omi W., Taniguchi T., Kaburaki T., Okajima M., Takamura M., Noda T., Ohta K., Itoh H., Goto Y., Kaneko S. (2008). The Attitudes of Japanese High School Students toward Cardiopulmonary Resuscitation. Resuscitation.

[14] Parnell M.M., Pearson J., Galletly D.C., Larsen P.D. (2006). Knowledge of and Attitudes towards Resuscitation in New Zealand High-School Students. Emerg. Med. J..

[15] Stroobants J., Monsieurs K.G., Devriendt B., Dreezen C., Vets P., Mols P. (2014). Schoolchildren as BLS Instructors for Relatives and Friends: Impact on Attitude towards Bystander CPR. Resuscitation.

[16] De Buck E., van Remoortel H., Dieltjens T., Verstraeten H., Clarysse M., Moens O., Vandekerckhove P. (2015). Evidence-Based Educational Pathway for the Integration of First Aid Training in School Curricula. Resuscitation.

[17] Lee S.A., Ju Y.J., Lee J.E., Hyun I.S., Nam J.Y., Han K.-T., Park E.-C. (2016). The Relationship between Sports Facility Accessibility and Physical Activity among Korean Adults. BMC Public Health.

[18] Skora J., Riegel B. (2001). Thoughts, Feelings, and Motivations of Bystanders Who Attempt to Resuscitate a Stranger: A Pilot Study. Am. J. Crit. Care.

[19] Lester C., Donnelly P., Weston C. (1997). Is Peer Tutoring Beneficial in the Context of School Resuscitation Training?. Health Educ. Res..

[20] Hubble M.W., Bachman M., Price R., Martin N., Huie D. (2003). Willingness of High School Students to Perform Cardiopulmonary Resuscitation and Automated External Defibrillation. Prehosp. Emerg. Care.

[21] Huang E.P.-C., Chiang W.-C., Hsieh M.-J., Wang H.-C., Yang C.-W., Lu T.-C., Wang C.-H., Chong K.-M., Lin C.-H., Kuo C.-W. (2019). Public Knowledge, Attitudes and Willingness Regarding Bystander Cardiopulmonary Resuscitation: A Nationwide Survey in Taiwan. J. Formos. Med. Assoc..

[22] De Smedt L., Depuydt C., Vekeman E., de Paepe P., Monsieurs K.G., Valcke M., Mpotos N. (2019). Awareness and Willingness to Perform CPR: A Survey amongst Flemish Schoolchildren, Teachers and Principals. Acta Clin. Belg..

[23] Pei L., Liang F., Sun S., Wang H., Dou H. (2019). Nursing Students’ Knowledge, Willingness, and Attitudes toward the First Aid Behavior as Bystanders in Traffic Accident Trauma: A Cross-Sectional Survey. Int. J. Nurs. Sci..

[24] Alomar M., Rouqi F.A., Eldali A. (2016). Knowledge, Attitude, and Belief Regarding Burn First Aid among Caregivers Attending Pediatric Emergency Medicine Departments. Burns.

[25] Alshammari K. (2021). Assessment of Knowledge, Attitude, and Practice about First Aid among Male School Teachers in Hail City. J. Fam. Med. Prim. Care.

[26] Mobarak A.S., Afifi R.M., Qulali A. (2015). First Aid Knowledge and Attitude of Secondary School Students in Saudi Arabia. Health.

[27] Petrovszki Z., Vári B., Győri F., Varga C., Molnár A. (2020). The Health Status and Physical Activity of Women Aged 45–54 and 55–64 in Szeged. Hunarian Rev. Sport Sci..

[28] Berki T., Tarjányi Z. (2022). The Role of Physical Activity, Enjoyment of Physical Activity, and School Performance in Learning Motivation among High School Students in Hungary. Children.

[29] Győri F., Berki T., Katona Z., Vári B., Katona Z., Petrovszki Z. (2021). Physical Activity in the Southern Great Plain Region of Hungary: The Role of Sociodemographics and Body Mass Index. Int. J. Environ. Res. Public Health.

[30] Molnár A., Boros-Balint I., Deak G.F., Andrei V.L., Ardelean V.P., Simonek J., Halmová N., Dobay B., Nagy Á.V., Vári B. Does the gross motor development of Romanian and Hungarian 6–7-year-old children depend on the degree of obesity? (First phase of a longitudinal study). Proceedings of the ICU 2019.

[31] Wang C., Chen P., Zhuang J. (2013). Validity and Reliability of International Physical Activity Questionnaire–Short Form in Chinese Youth. Res. Q. Exerc. Sport.

[32] Gabbe B.J. (2005). Incidence of Serious Injury and Death during Sport and Recreation Activities in Victoria, Australia. Br. J. Sports Med..

[33] Celse J., Nicolas M., Schilling P. (2017). Are Athletes More Cooperative than Nonathletes? A Laboratory Experiment. Manag. Decis. Econ..

[34] Gagnon A.G. (2016). Developmental Physical Education: How to Implement a Peer-Assistance Program to Help Low Performers. J. Phys. Educ. Recreat. Danc..

[35] Forero C.G., Maydeu-Olivares A., Gallardo-Pujol D. (2009). Factor Analysis with Ordinal Indicators: A Monte Carlo Study Comparing DWLS and ULS Estimation. Struct. Equ. Model. Multidiscip. J..

[36] Buckley L., Sheehan M., Dingli K., Reveruzzi B., Horgan V. (2021). Taking Care of Friends: The Implementation Evaluation of a Peer-Focused School Program Using First Aid to Reduce Adolescent Risk-Taking and Injury. Int. J. Environ. Res. Public Health.

[37] Zideman D.A., de Buck E.D.J., Singletary E.M., Cassan P., Chalkias A.F., Evans T.R., Hafner C.M., Handley A.J., Meyran D., Schunder-Tatzber S. (2015). European Resuscitation Council Guidelines for Resuscitation 2015 Section 9. First Aid. Resuscitation.

[38] Adib-Hajbaghery M., Kamrava Z. (2019). Iranian Teachers’ Knowledge about First Aid in the School Environment. Chin. J. Traumatol..

[39] Wang K.-M., Lin Y.-H., Huang Y.-C. (2012). The Knowledge and Attitude of Sports Injury Prevention and Management of Senior High School Athletes in Taiwan. Int. J. Sport Health Sci..

[40] Scott S.D., Hirschinger L.E., Cox K.R., McCoig M., Brandt J., Hall L.W. (2009). The Natural History of Recovery for the Healthcare Provider “Second Victim” after Adverse Patient Events. Qual. Saf. Health Care.

[41] Van der Graaff J., Branje S., de Wied M., Hawk S., van Lier P., Meeus W. (2014). Perspective Taking and Empathic Concern in Adolescence: Gender Differences in Developmental Changes. Dev. Psychol..

[42] Arasu S., Mathew S., Ramesh N., Fathima F., Johnson A. (2020). Safety First: Awareness and Attitude Regarding First Aid among College Students—A Cross-Sectional Study in Urban Bangalore. Int. J. Health Allied Sci..

[43] Kanstad B.K., Nilsen S.A.A., Fredriksen K. (2011). CPR Knowledge and Attitude to Performing Bystander CPR among Secondary School Students in Norway. Resuscitation.

[44] Mowlaie M., Besharat M.A., Pourbohlool S., Azizi L. (2011). The Mediation Effects of Self-Confidence and Sport Self-Efficacy on the Relationship Between Dimensions of Anger and Anger Control with Sport Performance. Procedia-Soc. Behav. Sci..

